# Radiographic area of large intestinal sand accumulation in horses may determine clinical significance

**DOI:** 10.1111/avj.70007

**Published:** 2025-08-08

**Authors:** IG Entwisle, DP Byrne, GD Lester, EJ McConnell

**Affiliations:** ^1^ Murdoch University Murdoch Western Australia Australia

**Keywords:** colic, magnesium sulphate, psyllium, radiographic area, sand enteropathy

## Abstract

**Background:**

Sand enteropathy is a common disease in horses worldwide. The presence of sand does not always cause disease. The amount of sand required to cause clinical disease is not well established.

**Objectives:**

To establish a weight‐indexed cut‐off for the diagnosis of clinically relevant sand enteropathy based on radiographic area.

**Study design:**

Cross‐sectional.

**Methods:**

Abdominal radiographs were acquired from clinically normal horses and compared with horses with clinical sand enteropathy. Sand area and sand area per kilogram of body weight were calculated. A receiver operating characteristic (ROC) curve was used to differentiate groups.

**Results:**

Seventy clinically normal horses and 57 sand enteropathy cases were included. Median sand area in control horses was 1 cm^2^ per 100 kg (interquartile range 0–6 cm^2^ per 100 kg), and in sand enteropathy horses was 118 cm^2^ per 100 kg (interquartile range 83–180 cm^2^ per 100 kg). Receiver operator characteristic (ROC) analysis revealed that using a cut‐off of 21 cm^2^ per 100 kg had a sensitivity of 98.25% (CI 90.71%–99.1%) and a specificity of 92.54% (CI 83.69%–96.77%) for a diagnosis of clinical sand enteropathy.

**Conclusions:**

A weight‐based cut‐off of >21 cm^2^ per 100 kg for the diagnosis of sand enteropathy was determined with excellent sensitivity and good specificity. This may aid in determining if the amount of sand accumulation in a horse is of clinical consequence.

Gastrointestinal abnormalities in horses associated with accumulation of sand in the large intestines of horses, referred to as sand enteropathy, are common in areas of sand‐rich soil worldwide. Colic is by far the most common clinical sign, followed by diarrhoea.^1^, ^2^, ^3^, ^4^, ^5^, ^6^, ^7^ The presence of sand alone, however, does not always cause clinical disease; various experimental methods of inducing sand enteropathy have failed to elicit consistent clinical signs, and small amounts of sand have been found incidentally both radiographically and in the faeces of clinically normal horses.^8^, ^9^, ^10^, ^11^

Radiographs are frequently used to evaluate the area of sand present, to aid in the diagnosis of sand enteropathy. Several radiographic scoring systems have been proposed, albeit they are largely subjective and have not been found to be consistently applicable prospectively.^4^, ^11^, ^12^, ^13^ More recent studies have used sand area in cm^2^, which provides a more quantitative assessment of sand burden.^5^, ^6^, ^14^ To date, only one study has investigated the presence of sand in clinically normal horses; however, it utilised one of the aforementioned scoring systems, and the control subjects were selected from a geographic area with a low incidence of sand enteropathy, and they did not consider the size of the horses.^11^

The aim of the current study was to investigate the area of sand present in both clinically normal horses and horses with sand enteropathy to determine a cut‐off point adjusted for body weight that can be used to determine the clinical significance of radiographic sand area. We hypothesised that the sand area per kilogram of body weight would be significantly smaller in clinically normal horses compared with horses presenting with clinical signs associated with sand enteropathy.

## Methods

The study was approved by the animal ethics committee of Murdoch University (permit number R3439/23). Clients with horses presenting to The Animal Hospital at Murdoch University between April 2023 and April 2024 that were undergoing sedation for nongastrointestinal disease were invited to participate. Signalment and history, including any gastrointestinal disease, were recorded, and cases were excluded if signs of colic within the last 6 months were reported. All horses underwent clinical examination before inclusion. Horses with diarrhoea on presentation were excluded. Those that met the selection criteria had left lateral radiographs of the cranioventral abdomen obtained. Dorsoventral radiographs were not able to be obtained due to body size. Radiographs were obtained using an x‐ray unit (M.T. Medical Technology CS01MS) and plate (XD 14 FXRD‐3643VAW) mounted on an overhead gantry and processed using ACGFA Musica Acquisition Workstation. The focal distance was set to 100 cm, and exposure settings were 100 kV/40 mAs for cranial views and 110 kV/64 mAs for more caudal views. In most horses, two to three radiographs were sufficient.

Clinical cases of sand enteropathy were retrieved from electronic medical records presenting to The Animal Hospital at Murdoch University between 2019 and 2023, and those with pretreatment radiographs included. Cases that underwent surgery were excluded. Radiographs were similarly standardised as left lateral views.

Radiographs were reviewed and measurement of sand area in cm^2^ calculated using OsiriX (Pixmeo) by use of the pencil region of interest (ROI) tool to trace around the outside of the sand area by the same observer. Where there were multiple small accumulations, the areas were added together to give a total in cm^2^. Body weight (kg) was obtained using electronic scales. An area per body weight was calculated by dividing the measured cm^2^ by body weight.

Normality was assessed by visual inspection of histograms, and non‐normally distributed data presented as medians with an interquartile range. Receiver operator characteristic (ROC) curves were plotted in GraphPad Prism 9.0 for an outcome of clinical sand enteropathy, to determine sensitivity and specificity of a cut‐off value to distinguish between the control group and the sand enteropathy group.

## Results

### Control horses

**Figure 1:**
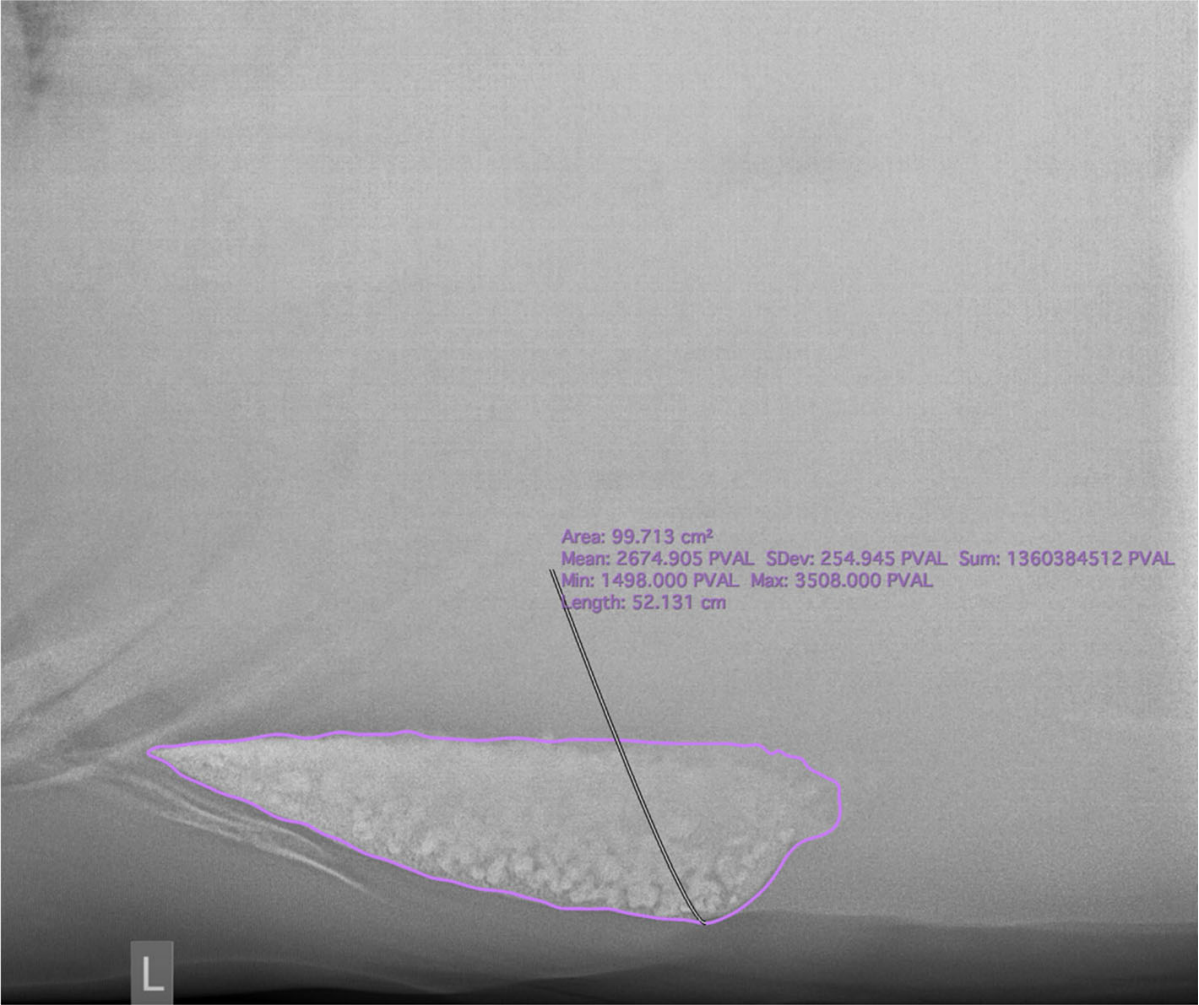
Left lateral radiograph of the cranioventral abdomen of a control horse showing one of the larger sand accumulations present in this group. Cranial is to the left. The sand area is 99.7cm^2^ as calculated using the pencil tool on Osirix, or 16 cm^2^ per 100 kg, which is below the weight‐adjusted cut‐off of 21cm^2^ per 100 kg.

The control group included 70 cases (4 Miniature Ponies, 6 Pony breeds, 18 Thoroughbreds, 13 Warmbloods, 7 Quarter Horses, 2 Draft breeds, 3 Arabians, 13 Standardbreds and4 Australian Stock Horses). Median age was 13 years (range 6–30 years) and median body weight was 540 kg (range 130–814 kg). Sand was present in 43/70 (61%) of clinically normal horses. Median sand area was 3.84 cm^2^ (interquartile range 0–10.1 cm^2^, range 0–253 cm^2^; see Figure 1), and median sand area per kg of body weight was 0.01 cm^2^/kg or 1 cm^2^ per 100 kg (interquartile range 0–0.06 cm^2^/kg, range 0–1.04 cm^2^/kg). Breeds with the highest sand areas per kg included 1 Miniature Pony (0.75 cm^2^/kg), 1 Warmblood (0.32 cm^2^/kg), 2 Thoroughbreds (0.25 cm^2^/kg & 0.24 cm^2^/kg) and 1 Pony breed (0.25 cm^2^/kg).

### Sand enteropathy horses

**Figure 2:**
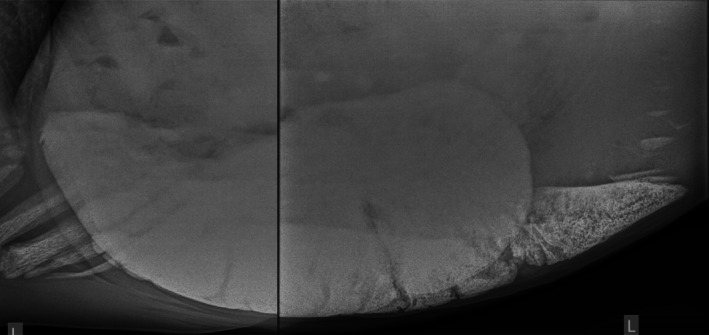
Left lateral composite radiograph of the cranioventral abdomen of a sand enteropathy horse showing one of the larger sand accumulations in this group. Cranial is to the left. The sand area is 739cm^2^ or 186cm^2^ per 100 kg, which is above the weight‐adjusted cut‐off of 21cm^2^ per 100 kg.

The sand enteropathy group included 57 cases (9 Miniature Ponies, 9 Pony breeds, 8 Thoroughbreds, 7 Warmbloods, 7 Quarter Horses, 4 Draft‐breeds, 4 Arabians, 2 Standardbreds, 2 Shetland Ponies and 2 Donkeys). Two horses presented for one and two repeat treatments, respectively. Median age was 12 years (range 10 months to 33 years) and median body weight was 430 kg (range 96–814 kg). Colic was the most common clinical sign reported (40/57; 70%), followed by weight loss or failure to gain weight (5/57; 9%) and diarrhoea (4/57; 7%). The median sand area on presentation was 465 cm^2^ (interquartile range 251–658 cm^2^, range 93–1276 cm^2^; see Figure 2), and median sand area per kg of body weight was 1.18 cm^2^/kg or 118 cm^2^ per 100 kg (interquartile range 0.83–1.80 cm^2^/kg, range 0.20–8.25 cm^2^/kg).

### Statistical analysis

**Figure 3:**
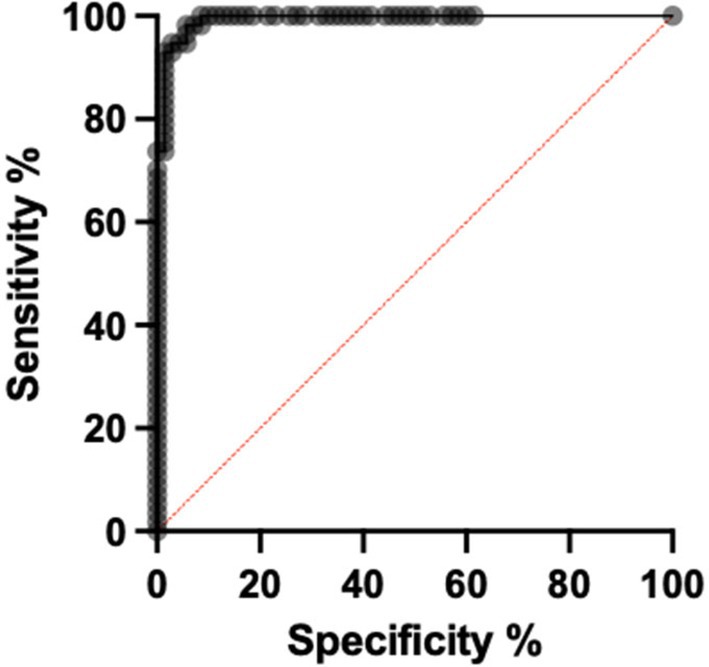
Receiver operating characteristic (ROC) curve discriminating sand enteropathy horses from clinically normal horses using sand area per kilogram of body weight. Area under the curve was 0.9935.

Receiver operator characteristic (ROC) of the area per kg revealed that using a cut‐off of 0.21 cm^2^/kg or 21 cm^2^ per 100 kg had a sensitivity of 98.25% (95% CI 90.71%–99.1%) and a specificity of 92.54% (95% CI 83.69%–96.77%) for a diagnosis of sand enteropathy, with a likelihood ratio of 13.16. Area under the curve for analysis was 0.9935 (95% CI 0.9849 to 1.0, P value < 0.0001; see Figure 3).

## Discussion

The findings of this study establish that a weight‐based cut‐off of greater than 21 cm^2^ per 100 kg can be used for the diagnosis of sand enteropathy with a high sensitivity (98.25%) and specificity (92.54%). The provision of a weight‐based cut‐off value may be more clinically applicable than previous radiographic scoring systems. Initial grading systems considered only the length and height of the large intestinal sand accumulation and did not consider the size of the horse.^13^ Keppie et al (2008) attempted to address this by using rib size as a ratio to the height and length of the sand burden, in addition to several other qualities, with those having a score of >7 having an 83% likelihood of a true diagnosis of sand colic.^12^ However, the controls used to develop this scoring system were not healthy, the sample size was small and subsequent investigators found parts of the scoring system to be inapplicable or scores <7 associated with colic signs.^4^, ^11^ With the improving quality of radiographs, it is now possible to estimate an area in cm^2^ which is more quantitative than previously reported scoring systems and has been adopted by several more recent studies.^5^, ^6^, ^14^ Using cm^2^/kg takes into account the size of the horse relative to the amount of sand. Our weight‐based cut‐off provides a clinical decision‐making tool using readily available methods for determining the relevance of radiographic sand burden.

Many clinically normal horses (61%) had small amounts of sand present with a median area of 3.84 cm^2^. This is consistent with a previous study that found sand on radiographs of control horses and supports that many horses in sand‐rich environments may have small amounts of sand present.^11^ Experimental methods of sand enteropathy, where several kilograms of sand have been administered, have failed to consistently elicit clinical signs.^8^, ^9^, ^10^ Thus, these findings support that the presence of sand alone does not always cause clinical disease. However, it has previously been difficult to determine based on radiographs what amount of sand can be considered significant. Based on our findings, up to 21 cm^2^ per 100 kg of sand on radiographs may be considered incidental and may not be the cause of gastrointestinal signs observed in clinically abnormal horses.

Our findings may also be useful in the prevention of sand enteropathy. Serial radiographs have previously been used to monitor treatment progress for sand elimination therapy.^1^, ^6^, ^14^, ^15^, ^16^ Radiographs can also be used for screening horses in areas with a high incidence of sand enteropathy, but previously it has been difficult to determine what amount of sand warrants treatment. Horses without current clinical disease but with areas of sand greater than 21 cm^2^ per 100 kg may be at risk of developing sand enteropathy, as the false‐negative rate of 2% is considered low. These horses could be considered candidates for sand elimination treatment, including in‐feed psyllium treatment or in‐hospital treatment with nasogastric administration of psyllium and magnesium sulphate.^6^, ^14^, ^15^ For horses with areas of sand below the cut‐off, regular screening radiographs with in‐feed psyllium may be sufficient management. Periodic screening radiographs combined with targeted treatment may help to prevent the development of sand enteropathy in horses that are considered to live in a high‐risk geographical area.

There are several limitations to this study. Firstly, although controls were excluded if they had a history of gastrointestinal disease, they were not followed prospectively, and thus, it is unknown if they went on to develop clinical disease. The control group and sand enteropathy were also not completely comparable; the largest difference was in the breed distribution, with more Miniature Ponies and Pony breeds in the sand enteropathy group. This is likely due to multiple causes. Miniature breeds have previously been described as being predisposed to the development of sand enteropathy.^4^ Most of the control group were also selected from horses presenting for musculoskeletal assessment, and Miniature Ponies are less likely to be used for performance and thus present for this type of assessment. It should therefore be noted that the weight‐associated cut‐offs may be less applicable to these breeds, where there was less representation in the control group. Radiographic density was also not evaluated in the study, which may also have an effect on the significance of an observed sand accumulation. Magnification of images may also have affected measurements. Further studies, including prospective application of this weight‐associated cut‐off, would be beneficial for determining its clinical utility.

## Conclusion

A weight‐based cut‐off of >21 cm^2^ per 100 kg for the diagnosis of sand enteropathy was determined with a sensitivity of 98.25% and specificity of 92.54%. This may aid in clinical decision making for determining if the amount of sand present on a radiograph is of clinical consequence, necessitating treatment.

## Conflicts of interest and sources of funding

The authors declare no conflicts of interest or sources of funding for the work presented here.

## Data Availability

The data that support the findings of this study are available from the corresponding author upon reasonable request.
